# Application of ozone gas for decontamination of nucleoside anticancer drugs

**DOI:** 10.1186/s40780-016-0058-3

**Published:** 2016-10-04

**Authors:** Ayumi Tsukamoto, Shunji Ishiwata, Asami Kajimoto, Ryusuke Murata, Rika Kitano, Tomomi Inoue, Takeshi Kotake

**Affiliations:** 1 Division of Medical Pharmaceutics & Therapeutics, Faculty of Pharmacy, Kindai University, 3-4-1 Kowakae, Higashi-Osaka, Osaka 577-8502 Japan; 2 Department of Pharmacy, Sakai City Medical Center, 1-1-1 Ebaraji-cho, Nishi-ku, Sakai City, 593-8304 Japan

**Keywords:** Ozone, Anticancer drugs, Occupational exposure, Gas, Decontaminant, Oxidative radical, Nucleoside

## Abstract

**Background:**

Exposure to anticancer drugs is hazardous and may lead to chromosomal abnormalities and spontaneous abortion in healthcare workers. Guidelines recommend surface decontamination and cleaning in order to minimize the occupational exposure to anticancer drugs, although no single process has been found to deactivate all currently available hazardous drugs. Ozone gas is oxidative and a decontaminant for bacteria; its characteristic as a gas has advantages in that it does not need to be wiped off or neutralized after use.

**Methods:**

The nucleoside anticancer drugs, cytarabine and fluorouracil, were exposed to ozone gas on plates under controlled humidity. The levels of exposed ozone were evaluated using the concentration-time (CT) value, which is the mathematical product of ozone concentration and exposure time. The effects of exposure to ozone on levels of the anticancer drugs were determined by high-performance liquid chromatography (HPLC).

**Results:**

The levels of cytarabine decreased with increasing CT value and were not detected beyond 40,000 CT. The decomposition levels of the anticancer drug by ozone were CT-dependent irrespective of the maximum concentration of ozone. Higher humidity in the range from 70 to 90 % accelerated the decomposition of cytarabine and fluorouracil, and neither of the drugs were detected at 90 % humidity after exposure to ozone gas.

**Conclusions:**

Ozone gas decomposed these nucleoside anticancer drugs. This is the first report of the applicability of ozone gas as a decontaminator for anticancer drugs.

## Background

Exposure to anticancer drugs may be hazardous to healthcare workers, since many of the anticancer drugs are carcinogenic and teratogenic, and cause reproductive toxicity, genotoxicity and/or organ toxicity [1]. These toxicities result in spontaneous abortions and chromosomal abnormalities in healthcare workers. Retrospective studies and meta-analysis revealed an association between exposure to chemotherapy and spontaneous abortion [2, 3]. The occurrences of chromosome 5 and 7 abnormalities in peripheral blood of healthcare workers handling the anticancer drugs were elevated compared to that in unexposed individuals [4].

In order to minimize the occupational exposure to anticancer drugs, National Institute of Occupational Safety and Health (NIOSH) Alert provided recommendations for routine cleaning, decontamination, housekeeping, and waste disposal [1]. The American Society of Health System Pharmacists (ASHP) guidelines on handling hazardous drugs recommended surface decontamination and cleaning using alcohol for disinfection, which does not deactivate any hazardous drugs and may result in the spread of contamination [5]. Although no single process has been found to deactivate all currently available hazardous drugs, wiping with sodium hypochlorite solution is recommended as a strong deactivator of many hazardous drugs followed by wiping with a detergent and thiosulfate neutralizer. However, areas that are not manually accessible, such as the plenum of a biological safety cabinet, cannot be cleaned by any wiping method.

Ozone is a colorless and strongly oxidative gas. It is a decontaminant for reduction of contamination with *Clostridium difficile* and *Bacillus subtilis* [6, 7]. Because of its characteristic as a gas, ozone has advantages, such as ability to reach anywhere, including inaccessible areas such as a plenum, and there is no need to remove or neutralize it after use.

This article reports on the applicability of ozone gas as a potential decontaminator for nucleoside anticancer drugs to prevent occupational exposure in healthcare workers. Relative humidity was also found to affect the oxidative activity of ozone against the named drugs.

## Methods

### Anticancer drugs

Cyloside N injection from Nippon Shinyaku Co., Ltd. and 5-fluorouracil (5-FU) injection from Kyowa Hakko Kirin Co., Ltd. were purchased commercially. Twenty mL of Cyloside N injection contained 40 mg of cytarabine, and 5 mL of 5-FU injection contained 250 mg of fluorouracil according to the package inserts.

### Exposure to ozone gas

Two-hundred micrograms of cytarabine or 500 μg of fluorouracil, was placed onto stainless-steel plates and kept at room temperature until dry. Ozone gas was generated by Handy Clean from Tamura Teco Co., Ltd. The plates containing the anticancer drugs were placed in an experimental chamber and exposed to ozone gas. Humidity in the chamber was determined by humidity sensor ES2HB (Omron, Kyoto Japan) and increased by ultrasonic humidifier Bottle Cube (Topland, Shizuoka Japan). Non-treated control plates were placed in another experimental chamber without exposure to ozone. The levels of exposure to ozone were evaluated as CT value (ppm•min), which is the mathematical product of ozone concentration (ppm) and exposure time (min). The exposure was performed under the condition in which ozone concentration and humidity was maintained at 35 ppm and 90 %, respectvitely and, CT was set at 80,000 unless otherwise described. After reaching an initially set CT value, ozone gas was inactivated with activated charcoal and the amount of anticancer drug remaining was analyzed by HPLC. All data were shown as mean of tripicte samples.

### HPLC analysis

A Capcell pak C18 MG II from Shiseido Co., Ltd was used as a column and the detection wavelength was 254 nm. The mobile phase for cytarabine and fluorouracil was 10 mmol/L phosphate buffer (pH 5.0):acetonitrile (95:5) and 50 mmol/L phosphate buffer (pH 5.0):methanol (85:15), respectively. The flow rate was 1 mL/min and the resulting chromatogram was analyzed using Chromato-PRO software from Run Time Corporation. The ozone-exposed and non-exposed control anticancer drugs on the plates were recovered by wiping the plates with cotton containing purified water. The reproducibility of HPLC and recovery rate of anticancer drug from the plate were examined prior to the ozone exposure experiment and was satisfactory for further experiments. The non-treated control levels of the anticancer drugs were estimated as 100 %.

## Results

**Fig. 1:**
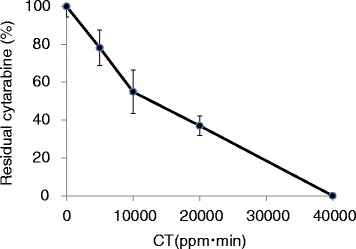
Effect of ozone gas on the levels of cytarabine. Data were mean of tripicte samples

**Fig. 2:**
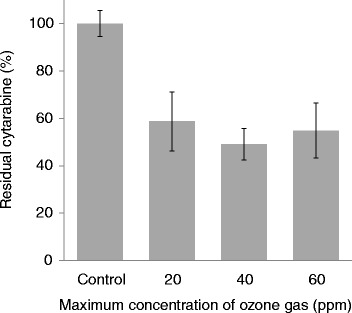
Effect of the maximum concentration of ozone gas on the levels of cytarabine. Data were mean of tripicte samples

To evaluate the effect of ozone gas on cytarabine, the stainless-steel plate was exposed to ozone in a chamber, in which the humidity was maintained at 90 %. The levels of cytarabine decreased with an increasing CT value and were not detected beyond 40,000 ppm•min (Fig. 1). Next, the effect of the maximum concentration of ozone on the anticancer drug was examined to reveal the usefulness of the CT value (Fig. 2). The exposure to ozone was terminated when the CT value reached 10,000 ppm•min during the experiment, and the maximum concentration of ozone was set at 20, 40, and 60 ppm. The levels of cytarabine decreased to approximately 50 % after exposure to ozone, and no differences were detected between the groups irrespective of the maximum concentration of ozone. The effect of the humidity on the levels of cytarabine during exposure to ozone is shown in Table 1. The level of cytarabine at 70 % humidity was 88.2 % compared to that observed for the non-treated control group. At 80 % humidity, the levels of cytarabine decreased to 2.9 % compared with control and it was undetectable at 90 % humidity. Levels of fluorouracil after exposure to ozone under various humidity conditions are shown in Table 2. Ozone had no effect on the level of fluorouracil at 70 % humidity; however, the level of fluorouracil at 80 % humidity was 13 % compared to that in control, and was undetectable at 90 % humidity.

**Table 1:** Effect of humidity on cytarabine during exposure of ozone gas

Humidity (%)	Ozone exposure	Residual cytarabine (%)				
					Mean	SD
70	-	100.9	98.6	100.5	100.0	1.2
	+	88.6	87.5	88.7	88.2	0.7
80	-	100.6	100.3	99.1	100.0	0.8
	+	3.3	2.9	2.5	2.9	0.4
90	-	100.6	98.4	101.0	100.0	1.4
	+	0.0	0.0	0.0	0.0	0.0

**Table 2:** Effect of humidity on fluorouracil during exposure of ozone gas

Humidity (%)	Ozone exposure	Residual fluorouracil (%)				
					Mean	SD
70	-	92.0	102.3	105.7	100.0	7.2
	+	101.7	110.1	111.1	107.6	5.2
80	-	93.8	94.0	112.2	100.0	10.6
	+	0.0	22.1	16.9	13.0	11.6
90	-	96.6	98.3	105.1	100.0	4.5
	+	0.0	0.0	0.0	0.0	0.0

## Discussion

Many kinds of approaches, such as the use of closed systems, are recommended to prevent the occupational exposure of healthcare workers to anticancer drugs [1, 5]. However, once contamination has occurred, anticancer drugs spread to the environment and must be cleared thereafter. Spill kits, which contain solutions of sodium hydroxide, sodium thiosulfate, detergent, and/or sodium hypochlorite, are usually recommended for decontamination. Mochizuki et al. [8] showed that paclitaxel and docetaxel were decomposed by wiping with sodium hydroxide solution while cyclophosphamide, fluorouracil, and gemcitabine were not. Therefore, no single process has been found to deactivate all anticancer drugs to date. In addition, areas that are not accessible by hand such as a plenum in a biological safety cabinet, or areas healthcare workers do not expect to be contaminated, could not be cleared off using a wiping method.

Ozone gas is oxidative and has several presumed advantages when used as a deactivator of the anticancer drugs. It has a high redox potential and will accomplish disinfection in addition to decomposing the anticancer drugs. Ozone, being a gas, can be used to decontaminate large areas and reach inaccessible locations, and does not rely on workers identifying surfaces for decontamination. Since ozone gas can be generated on-site from oxygen in the air, there are fewer safety and economic problems associated with shipping and storage. Residual ozone gas can be also easily decomposed by activated charcoal after the deactivation of the anticancer drug is complete. On the other hand, in Occupational Safety & Health Administration (OSHA), part of the United States Department of Labor, guidelines for ozone, ozone levels should never exceed 0.1 ppm for 8 h per day exposure in the workplace [9]. The detection limit of ozone gas in human is approximately 0.01 ppm because of its unique smell, which help the workers notice and tackle the toxicity of the gas before they suffer health hazard. However, instruction and careful handling is needed. Ozone gas is also reactive and corrosive, thus requiring the use of a corrosion-resistant material such as stainless steel.

It has been shown here that ozone gas decomposes the nucleoside anticancer drugs cytarabine and fluorouracil. The decomposition by ozone is CT-dependent and not dependent on the maximum concentration of ozone. The decomposition by ozone is also time-dependent if the concentration of ozone is maintained constant through the exposure. In addition, higher humidity accelerates decomposition of the anticancer drugs. Ozone is converted to hydroxyl radicals in the presence of water, which has higher redox potential and reactivity than ozone [10], and the elevated levels of humidity might facilitate the oxidative reactions of the anticancer drugs.

The results reported here suggest that ozone gas can be a candidate for a decontaminator of anticancer drugs. Ozone gas has advantages in deactivation of the anticancer drugs described above. This is the first report of the cleanup effects of ozone gas on the nucleoside anticancer drugs to the best of the authors’ knowledge. Further research into the applicability of ozone as a decontaminator for anticancer drugs is planned, while further studies on the activity of ozone with other anticancer drugs are needed.

## Conclusion

It has been demonstrated that ozone gas decomposed the investigated nucleoside anticancer drugs in a CT-dependent and humidity-dependent manner. The applicability of ozone gas as a decontaminator for anticancer drugs can be suggested, since ozone gas has some advantages in that it can access any site, be generated on-site from air, and easily deactivated.

## Abbreviations

ASHP: The American Society of Health System Pharmacists
CT: Concentration-time
HPLC: High-performance liquid chromatography
NIOSH: National Institute of Occupational Safety and Health
OSHA: Occupational Safety & Health Administration

## References

[1] National Institute for Occupational Safety and Health (2004). NIOSH alert: preventing occupational exposure to antineoplastic and other hazardous drugs in health care settings.

[2] Lawson CC, Rocheleau CM, Whelan EA, Lividoti Hibert EN, Grajewski B, Spiegelman D (2012). Occupational exposures among nurses and risk of spontaneous abortion. Am J Obstet Gynecol.

[3] Dranitsaris G, Johnston M, Poirier S, Schueller T, Milliken D, Green E (2005). Are health care providers who work with cancer drugs at an increased risk for toxic events? A systematic review and meta-analysis of the literature. J Oncol Pharm Pract.

[4] McDiarmid MA, Oliver MS, Roth TS, Rogers B, Escalante C (2010). Chromosome 5 and 7 abnormalities in oncology personnel handling anticancer drugs. J Occup Environ Med.

[5] American Society of Health-System Pharmacists (2006). ASHP guidelines on handling hazardous drugs. Am J Health-Syst Pharm.

[6] Davies A, Pottage T, Bennett A, Walker J (2011). Gaseous and air decontamination technologies for Clostridium difficile in the healthcare environment. J Hosp Infect.

[7] Aydogan A, Gurol MD (2006). Application of gaseous ozone for inactivation of bacillus subtilis spores. J Air Waste Manage Assoc.

[8] Mochizuki C, Fujikawa I, Tei G, Yoshida J (2008). A comparison of cleaning solutions in the biological safety cabinet for preparation of anti-cancer agents. J Jpn Soc Hosp Pharm.

[9] Occupational Safety and Health Administration, United States of Labor: Ozone. https://www.osha.gov/dts/chemicalsampling/data/CH_259300.html. Accessed 2 Sep 2016.

[10] United States Environmental Protection Agency (1999). Alternative disinfectants and oxidants guidance manual.

